# Prevalence of Dyslipidemia Among Antiretroviral-Naive HIV-Infected Individuals in China

**DOI:** 10.1097/MD.0000000000002201

**Published:** 2015-12-07

**Authors:** Yinzhong Shen, Jiangrong Wang, Zhenyan Wang, Tangkai Qi, Wei Song, Yang Tang, Li Liu, Renfang Zhang, Hongzhou Lu

**Affiliations:** From the Department of Infectious Disease, Shanghai Public Health Clinical Center, Fudan University, Shanghai, China (YS, JW, ZW, TQ, WS, YT, LL, RZ, HL).

## Abstract

Little is known about the epidemiological features of dyslipidemia among antiretroviral-naive HIV-infected individuals in China. We used a cross-sectional study design to estimate the prevalence of dyslipidemia in this population, and to identify risk factors associated with the presence of dyslipidemia.

One thousand five hundred and eighteen antiretroviral-naive HIV-infected individuals and 347 HIV-negative subjects in China were enrolled during 2009 to 2010. Demographics and medical histories were recorded. After an overnight fast, serum samples were collected to measure lipid levels. Factors associated with the presence of dyslipidemia were analyzed by logistic regression.

Mean total cholesterol (TC), low-density lipoprotein cholesterol (LDL), high-density lipoprotein cholesterol (HDL) levels were lower in HIV-positive than HIV-negative subjects, but mean triglyceride (TG) was higher in HIV-positive subjects. The overall prevalence of dyslipidemia in HIV-positive and HIV-negative groups did not differ (75.6% vs. 73.7%, *P* = 0.580). However, the prevalence of high TC (8.4% vs. 28.2%, *P* < 0.001) and high LDL (8.5% vs. 62.6%, *P* < 0.001) was lower in HIV-positive than HIV-negative subjects, and the prevalence of high TG (33.9% vs. 17.0%, *P* < 0.001) and low HDL (59.6% vs. 11.2%, *P* < 0.001) was higher in HIV-positive than HIV-negative subjects. Logistic analysis showed that HIV positivity was significantly associated with both an increased risk of high TG and low HDL and a decreased risk of high TC and high LDL. The mean levels of TC, of LDL and of HDL showed an increasing trend with increasing CD4 count in HIV-positive subjects. Multivariable logistic regression found that lower CD4 count was significantly associated with both an increased risk of high TG and low HDL and a decreased risk of high TC in HIV-positive subjects.

Among antiretroviral-naive HIV-infected Chinese adults, there was a high prevalence of dyslipidemia characterized by high TG and low HDL, which was associated with lower CD4 counts. These data support the assessment of lipid profiles before and after initiation of antiretroviral therapy regardless of age.

## INTRODUCTION

Combination antiretroviral therapy (ART), widely used in clinical practice, has dramatically reduced HIV-associated morbidity and mortality and has turned HIV infection into a manageable chronic disease.^1,2^ However, metabolic abnormalities and cardiovascular diseases have been reported with increasing frequency and have been an important cause of mortality and morbidity in the HIV-infected population.^2–4^ Individualized approaches, taking into consideration quality-of-life issues, and assessment of potential cardiovascular risks, are now an important component of effective care of HIV-infected patients.^5^

Lipid abnormalities are common with HIV infection and its treatment is a prevalent condition.^6–8^ Dyslipidemia in HIV-infected individuals includes decreased high-density lipoprotein cholesterol (HDL) and low-density lipoprotein cholesterol (LDL) concentrations, followed by increased triglycerides (TG) and very low-density lipoprotein cholesterol (VLDL) levels in later-stage of disease.^7^ After ART, TG, total cholesterol (TC), LDL can be increased. In particular, protease inhibitors (PI) are associated with high TC, high blood sugar, as well as the emergence of somatic obesity.^8^

The prevalence of dyslipidemia is likely to increase with the aging of the HIV-positive population due to ART, and differs by demographic characteristics, lifestyle, antiretroviral exposure, and region.^6,9–12^ AIDS is a major public health problem in China. Our previous study showed that hyperglycemia is highly prevalent among Chinese adults with newly diagnosed HIV/AIDS.^13^ Thus far, the epidemiological features of dyslipidemia among antiretroviral-naive HIV-infected individuals in China have not been extensively studied. The present study aimed to estimate the prevalence of dyslipidemia among this population, and to identify risk factors associated with the presence of dyslipidemia.

## METHODS

### Study Population

This was a cross-sectional study. The survey was conducted in 10 provinces and municipalities in China between 2009 and 2010, including Xinjiang, Jiangxi, Henan, Heilongjiang, Guangdong, Shaanxi, Guangxi, Hunan, Shanghai, and Yunnan. The details of the study HIV-infected population have been described previously.^13^ Subjects aged 18 years or more at the time of enrolment with confirmed HIV infection and naive to antiretrovirals were included in this study.

We also enrolled 347 HIV-negative Chinese subjects to serve as controls. People who received physical examination at Shanghai Public Health Clinical Center were enrolled. Subjects aged 18 years or more at the time of enrolment without HIV infection were eligible for this study group. All subjects were confirmed to be negative for HIV antibody through laboratory detection.

### Blood Samples and Measurements

Blood sample was collected from each participant after at least 12 hours of overnight fasting, for the measurement of plasma lipid levels. Plasma lipid level was measured by an automated chemistry analyzer, at the clinical biochemical laboratories in each province. All the laboratories completed a standardization and certification program.

### Definitions

Conditions were defined as follows: high TC (TC ≥ 200 mg/dl [5.2 mmol/L]), high TG (TG ≥ 150 mg/dl [1.7 mmol/L]), high LDL (LDL ≥ 130 mg/dl [3.37 mmol/L]), and low HDL (HDL < 40 mg/dl [1.04 mmol/L]). Dyslipidemia was defined as either high TC, high TG, high LDL, or low HDL. Dyslipidemia was assessed according to the United States National Cholesterol Education Program, Adult Treatment Panel (NCEP-ATP) III guidelines.^14^ The definitions for dyslipidemia used in China are similar to NCEP-ATP III criteria.

### Data Collection

Detailed descriptions of data collection and study methodology have been reported previously.^13^ For all participants, data were collected on demographics, risk-behavior information, and laboratory test results. Study variables included age, sex, ethnicity, hepatitis C virus (HCV) serostatus, HIV status, HIV transmission route, and CD4 cell counts.

### Statistical Analysis

Data were entered and analyzed using IBM SPSS Statistics version 19. Categorical variables were presented as percentages with frequency while continuous variables were presented as mean and standard deviation (SD). Pearson Chi-square test was applied for categorical attributes, continuous variables were compared using *t* tests or One-way ANOVA. Correlations between plasma lipid and CD4 count were evaluated using the Pearson correlation test. Multivariate logistic regression models were used to analyze the association between dyslipidemia and relevant baseline variables. Multivariable models were fitted using backward stepwise selection and variables with *P* < 0.10 being included in the models. Variables with multiple categories were included if overall tests for trend in heterogeneity were significant (*P* < 0.10). A 2-tailed *P* < 0.05 was considered statistically significant.

### Ethics Statement

The study was approved by the Shanghai Public Health Clinical Center Ethics Committee. Written informed consent was given by all participants.

## RESULTS

### Study Population Characteristics

We included a total of 1865 Chinese adults in this study; 1518 (81.4%) were newly diagnosed HIV-infected patients, 347 (18.6%) were HIV-negative individuals. The study subject was primarily male (66.1%), with a median age of 38 years old. Most subjects were of Han ethnicity (79.8%), 20.2% were ethnic minorities.

Among the 1518 HIV-positive adults, 83 patients were from Xinjiang, 19 from Jiangxi, 24 from Henan, 35 from Heilongjiang, 76 from Guangdong, 77 from Shaanxi, 416 from Guangxi, 175 from Hunan, 261 from Shanghai, and 352 from Yunnan. The HIV-infected subjects were primarily male (75.1%), the median age was 38 years, 24.8% were ethnic minorities, 14.9% were positive for HCVAb, and the median CD4 count was 92 cells/mm^3^. Most HIV-positive subjects acquired HIV through sexual contact (71.2%).

All the 347 HIV-negative adults were from Shanghai and of Han ethnicity, all were negative for HCVAb. The study HIV-negative subjects were primarily female (73.5%), with median age of 37 years.

### Serum Lipid Levels Among the Study Population

The serum lipid levels of the study subjects are shown in Table 1. Mean TC, LDL, and HDL were significantly lower in HIV-positive than HIV-negative subjects, while mean TG was significantly higher in HIV-positive than HIV-negative subjects. Both TC and LDL differed by ethnicity, sex, age, and HCV serostatus. HDL differed by ethnicity, sex, and HCV serostatus. TG differed by sex.

**TABLE 1:**
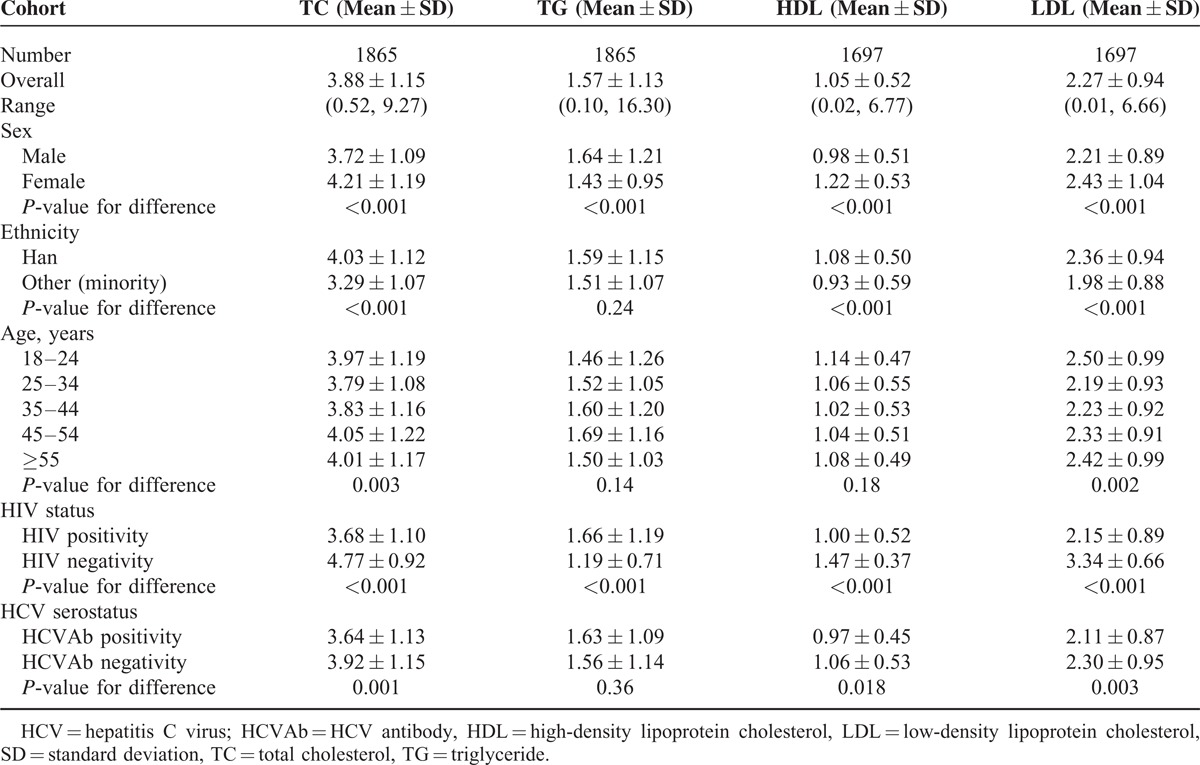
Lipid Levels Among All Study Subjects

### Prevalence of Dyslipidemia Among the Study Population

The overall prevalence of dyslipidemia among the study population was 75.4%. The overall prevalence of dyslipidemia did not differ significantly between HIV-positive and HIV-negative subjects (75.6% and 73.7%, respectively, *P* = 0.58). The prevalence of dyslipidemia among the study population is shown in Table 2. Overall, the prevalence of high TC, high TG, high LDL, and low HDL were 12.1%, 30.7%, 14.2%, and 54.4%, respectively (Table 2). Prevalence of high TC, high TG, high LDL, and low HDL were 8.4%, 33.9%, 8.5%, and 59.6% among HIV-positive subjects and 28.2%, 17.0%, 62.6%, and 11.2% among HIV-negative subjects, respectively. The prevalence of high TC and high LDL was lower in HIV-positive than HIV-negative subjects (*P* < 0.001, *P* < 0.001); whereas, the prevalence of high TG and low HDL was higher in HIV-positive than HIV-negative subjects (*P* < 0.001, *P* < 0.001). Univariate analysis showed that high TC was also associated with ethnicity, sex, and age; high TG was associated with sex and age; both high LDL and low HDL were associated with sex, ethnicity, and HCV serostatus.

**TABLE 2:**
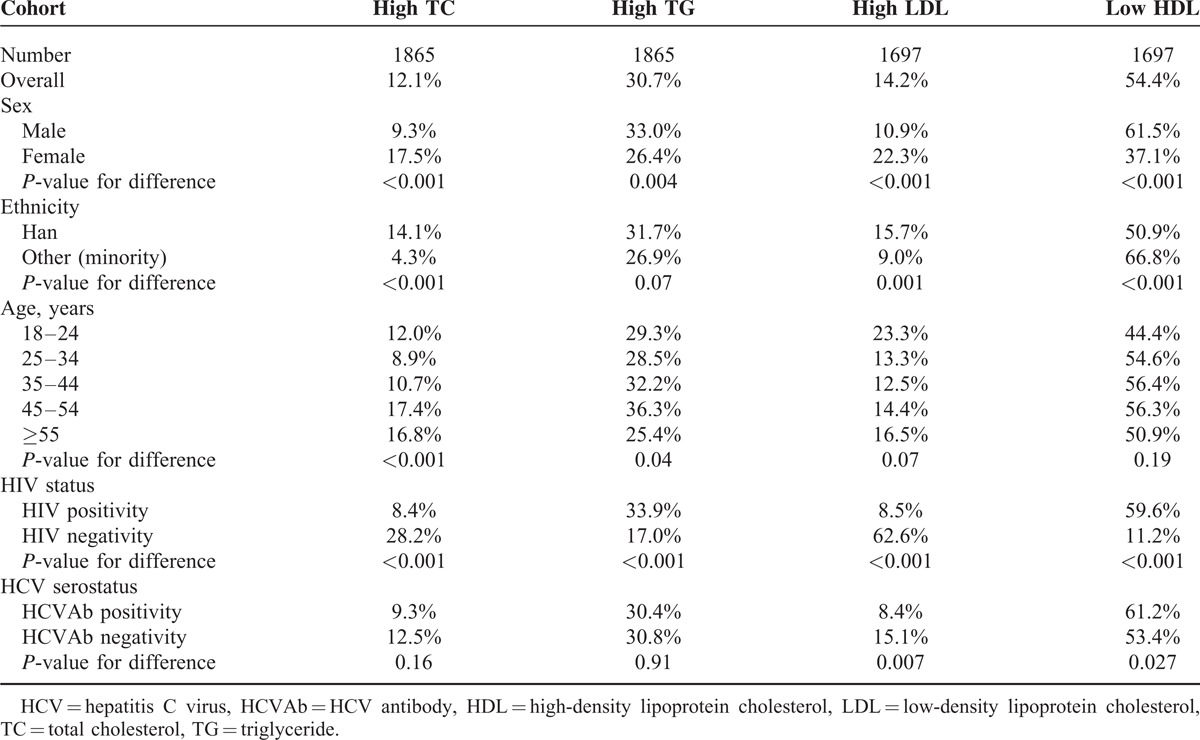
Prevalence of Dyslipidemia Among All Study Subjects

### Risk Factors for Dyslipidemia Among the Study Population

In a multivariate analysis using a logistic regression model, we analyzed factors associated with the presence of dyslipidemia among the study population (including sex, age, ethnicity, HCV serostatus, and HIV status). Table 3 summarizes the results of the final regression models. Older age was significantly associated with an increased risk of high TC, minority ethnicity, and HIV infection were significantly associated with a decreased risk of high TC; HIV infection was significantly associated with an increased risk of high TG, minority ethnicity was a protective factor for high TG; HIV infection was significantly associated with a decreased risk of high LDL; minority ethnicity and HIV infection were significantly associated with an increased risk of low HDL, female sex is a protective factor for low HDL. HCV serostatus failed to show an association with the presence of dyslipidemia.

**TABLE 3:**
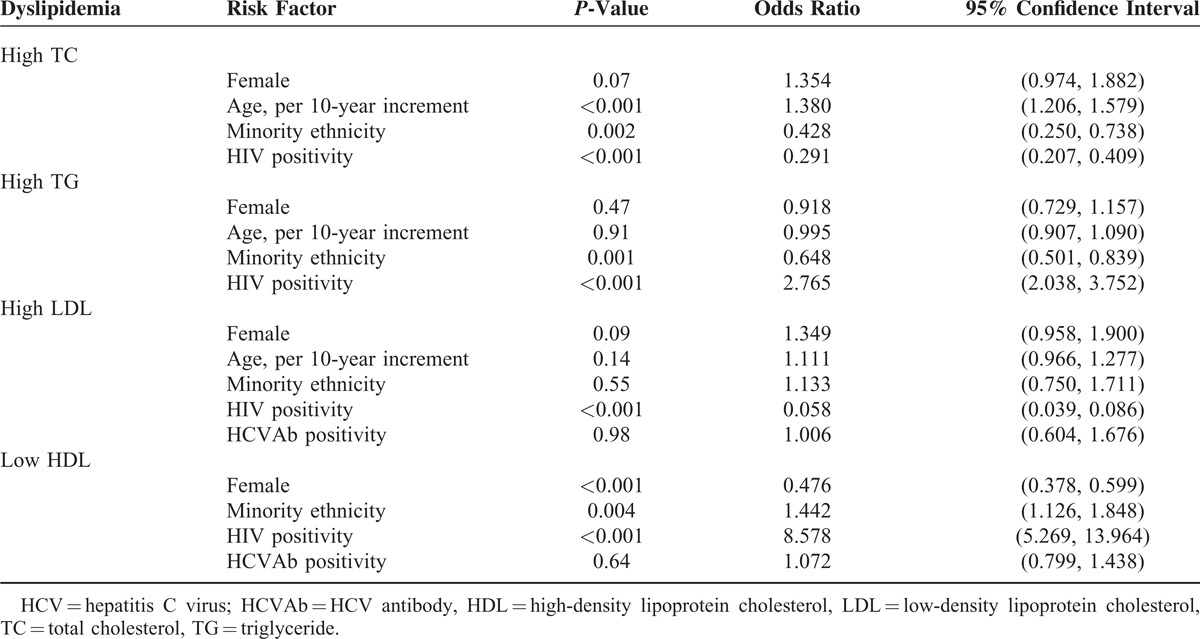
Identification of Risk Factors for the Presence of Dyslipidemia Among All Study Subjects, Results of the Regression Models

### Serum Lipid Levels Among HIV-Positive Subjects

The serum lipid levels among HIV-positive subjects are shown in Table 4. TC differed by sex, ethnicity, age, CD4 count, and HIV transmission route; TG differed by ethnicity and HIV transmission route; LDL differed by ethnicity, age, CD4 count, and HIV transmission route; HDL differed by sex, ethnicity, and CD4 count. Serum lipid levels did not differ significantly according to HCV serostatus.

**TABLE 4:**
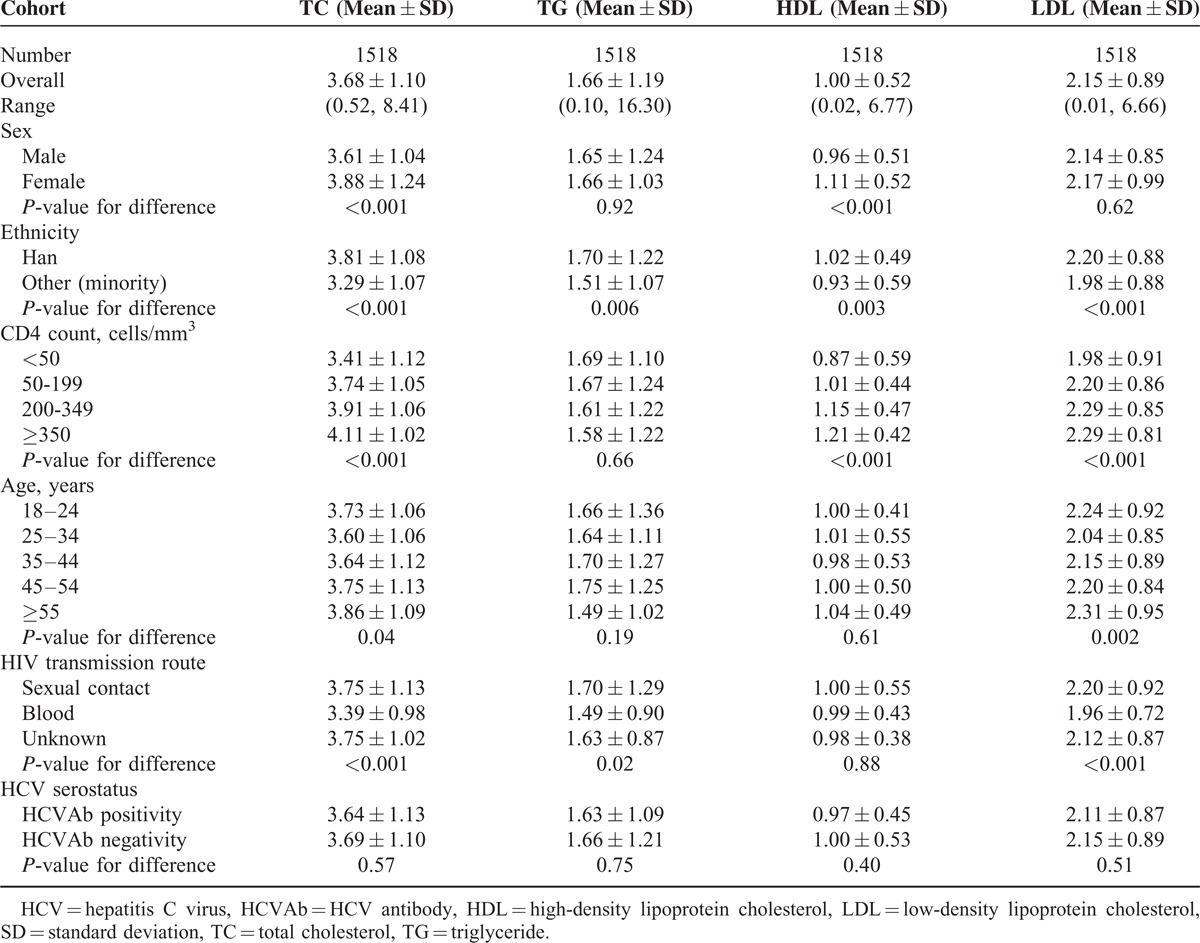
Lipid Levels Among HIV/AIDS Subjects

The mean levels of TC, LDL, and HDL showed an increasing trend with increasing CD4 count (*P* < 0.001 for TC, LDL, and HDL). The mean levels of TG showed a decreasing trend with increasing CD4 count, but the difference was not statistically significant (*P* = 0.66). Using the Pearson correlation, there was a significant and positive correlation between TC level (r^2^ = 0.045; *P* < 0.001), LDL level (r^2^ = 0.018; *P* < 0.001), HDL level (r^2^ = 0.053; *P* < 0.001), and CD4 count, respectively.

### Prevalence of Dyslipidemia Among HIV-Positive Subjects

Univariate analysis showed that high TC was associated with Han ethnicity, female sex, higher CD4 count, and transmission by sexual contact; high TG was associated with Han ethnicity and younger age; high LDL was associated with transmission by sexual contact; low HDL was associated with ethnicity minority, male sex, and lower CD4 count (Table 5). The prevalence of high TC increased with increasing CD4 count (*P* = 0.01), the prevalence of low HDL decreased with increasing CD4 count (*P* < 0.001). The prevalence of dyslipidemia did not differ significantly according to HCV serostatus.

**TABLE 5:**
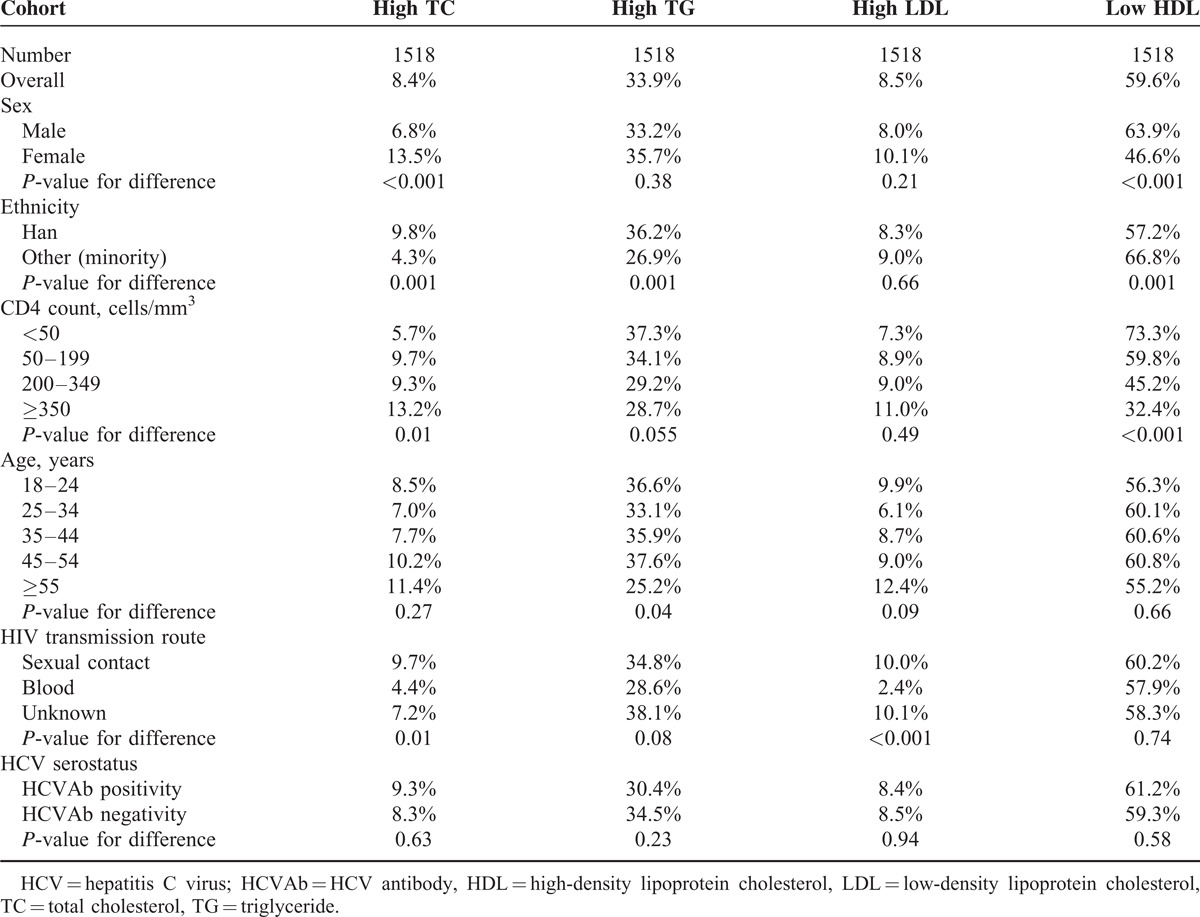
Prevalence of Dyslipidemia Among HIV/AIDS Subjects

### Risk Factors for Dyslipidemia Among HIV-Positive Subjects

In a multivariate analysis using a logistic regression model, we analyzed factors associated with the presence of dyslipidemia among HIV-positive subjects, including sex, age, ethnicity, HIV transmission route, CD4 count, and HCV serostatus (Table 6). Female sex and higher CD4 count were significantly associated with an increased risk of high TC, minority ethnicity, and transmission by blood were significantly associated with a decreased risk of high TC; minority ethnicity and higher CD4 count were a protective factor for high TG; transmission by blood was significantly associated with a decreased risk of high LDL; female sex and higher CD4 count were a protective factor for low HDL.

**TABLE 6:**
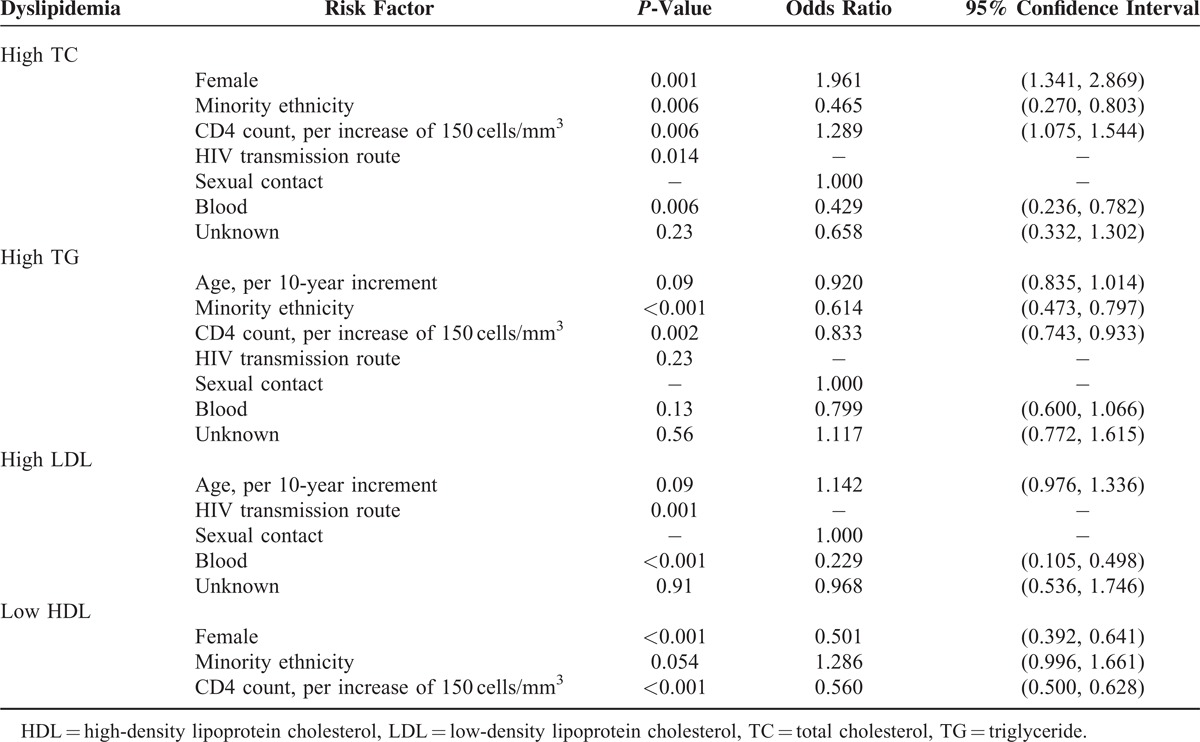
Identification of Risk Factors for the Presence of Dyslipidemia Among HIV/AIDS Subjects, Results of the Regression Models

## DISCUSSION

The importance of diagnosis and management of dyslipidemias in HIV-infected individuals is increasingly recognized. This study was designed to investigate the prevalence and associated factors of dyslipidemia among ART-naive HIV-infected individuals in China. To our knowledge, this is the first survey to document the epidemiological features of dyslipidemia in this population in China.

Our study showed that antiretroviral-naive HIV-infected individuals had a variety of lipid abnormalities including significant elevation of TG and reduced level of TC, LDL, and HDL compared with HIV-negative subjects. The same pattern of lipid abnormalities was previously observed in other ART-naive HIV populations.^6,15–18^ However, a case–control study conducted in Nigeria found that antiretroviral-naive HIV positive patients had significant elevation in LDL, reduction in HDL and TC compared with HIV negative controls.^9^ A cross-sectional substudy of the Women's Interagency HIV Study found that antiretroviral-naive HIV-positive women had lower HDL and higher TG but not lower LDL than HIV-negative women.^10^ In Uganda, reports showed that rural Ugandans with advanced HIV disease have infrequent elevations in serum TC, LDL, and TG before ART.^19^ These findings reinforce results from our study, which show that antiretroviral-naive HIV-infected individuals exhibit a wide range of dyslipidemias.

Our data showed that the overall prevalence of dyslipidemia among HIV-positive individuals was as common as among HIV-negative controls. However, the prevalence of high TC and LDL was lower in HIV-positive individuals than HIV-negative subjects, and the prevalence of high TG and low HDL was higher in HIV-positive than HIV-negative subjects. Based on these data, we conclude that high TG and low HDL are highly prevalent among antiretroviral-naive HIV-infected Chinese adults, and that high TC and high LDL are less common in this population. Our data indicate that dyslipidemias are very common and an important health problem among antiretroviral-naive HIV-infected individuals in China. To control dyslipidemias, effective public health education and urgent measures are essential. Efforts to promote screening, risk stratification, and initiating appropriate treatment should be intensified. Newly diagnosed HIV/AIDS patients should be screened for dyslipidemia and this should become an important part of HIV care. In China, routine screening for dyslipidemia is already recommended in HIV-infected patients both before and after initiating HIV treatment. Given the high prevalence of dyslipidemia, cardiovascular disease risk prevention counseling and management should be integrated into HIV care in China.

The results of our study are comparable to some studies in other countries, which also indicate a high prevalence of dyslipidemia in ART-naive HIV-infected adults and children. A study conducted between November 2004 and June 2008 in Tanzania showed that low HDL was prevalent in 67% and increased TG in 28% of the ART-naive HIV-infected patients.^6^ An investigation conducted in Ethiopia revealed that 76.9% of pre-ART patients had at least 1 laboratory abnormality, which is compatible with a diagnosis of dyslipidemia.^20^ Another study conducted in 274 HIV-infected ART-naive Thai and Cambodian children 1 to 12 years of age with CD4 between 15% and 24% showed that overall 63.9% had dyslipidemia with high TG and low HDL being the most common: 28% and 45%, respectively, while 2% had high TC or high LDL.^11^

Our study showed that infection with HIV was significantly associated with dyslipidemia. Prior studies have shown evidence for HIV to be associated with dyslipidemia.^10,12,15^ Two studies have explored how much the HIV infection and/or treatment contribute to the changes in HDL levels. The results suggest that hypoalphalipoproteinemia in patients with HIV is likely to be secondary to HIV infection itself.^16,18^ The abnormal lipid metabolism that occurs during the course of ART for HIV has gained much attention in recent years. Dyslipidemia is very common in HIV-infected patients receiving ART.^2^ Dyslipidemia among HIV patients, although multifactorial, has been associated with HIV infection itself, as well as the use of antiretroviral agents.^21^ It has been demonstrated that patients on ART are at increased risk for developing metabolic abnormalities that include elevated levels of TG and LDL and reduced levels of HDL.^20,22,23^ A study based on the 6 years follow-up of 57 Chinese HIV-infected patients starting ART was conducted in Hunan province. The patients were on nevirapine-based regimens with a healthy lipids profile. Dyslipidemia developed in 26.3% of the patients with high TC and high TG after 5 to 6 years of follow-up. These data support the assessment of lipid profiles before and after initiation of ART in China.^24^

Our study showed that the mean levels of TC, LDL, and HDL increased with increasing CD4 count in HIV positive patients. Multivariable logistic regression found that lower CD4 count was significantly associated with both an increased risk of high TG and low HDL and a decreased risk of high TC in HIV-infected patients. These results indicate that dyslipidemia has a strong correlation with HIV disease stage. The strong correlation between dyslipidemia with HIV disease progression has also been observed in other treatment-naive populations in other studies.^6,12,16^ These findings strengthen recommendations for earlier ART initiation.

In agreement with other studies,^9,12^ both demographic and HIV-related factors are associated with dyslipidemia. In our sample, dyslipidemia was significantly associated with the factors of sex (only for high TC and low HDL), ethnicity (only for high TC and high TG), and HIV transmission route (only for high TC and high LDL). HCV serostatus and age failed to show association with dyslipidemia. These findings provide focused targets for improving routine screening for dyslipidemia in order to early stratify cardiovascular disease risk and initiation of appropriate treatment.

This study has some limitations. First, the cross-sectional study design limited the ability to assess causal relationships between the risk factors and dyslipidemia. Second, potential sample selection bias may have affected the findings. There was imbalance with regards to sex and ethnicity in the HIV-positive and HIV-negative subjects, and there were no minorities in the control group. In order to solve this issue, we used multiple logistic regression models to adjust for the effects of potential confounders. This study is the largest epidemiological survey of dyslipidemia in patients newly diagnosed with HIV/AIDS in China to date. The big sample size can help minimize bias. Third, some studies showed that body-mass index, lifestyle, smoking, family history of dyslipidemia, and HIV viral load are associated with dyslipidemia,^6,9,12,25–27^ but these factors were not investigated in this study. Therefore, the association between these factors and dyslipidemia cannot be assessed. Further study involving more potential risk factors is needed. In addition, we did not collect data on use of lipid-lowering therapy in this study, although lipid-lowering therapy is uncommonly prescribed in this age group, this may have led to an underestimation of the true prevalence of dyslipidemia. Despite these limitations, the present study provides significant data on the epidemiological features of dyslipidemia in ART-naive HIV-infected Chinese adults.

In conclusion, among antiretroviral-naive HIV-infected Chinese adults, there was a high prevalence of dyslipidemia characterized by high TG and low HDL, which was associated with lower CD4 counts. Our data support screening for dyslipidemia in HIV-infected individuals before and after initiating HIV treatment regardless of age. Further research is needed to study the impact of different lipid abnormalities on the prognosis and quality of life of patients with HIV/AIDS.
